# Bioinspired Surfaces With Switchable Wettability

**DOI:** 10.3389/fchem.2020.00692

**Published:** 2020-08-12

**Authors:** Dong-Dong Han, Qing Cai, Zhao-Di Chen, Ji-Chao Li, Jiang-Wei Mao, Pin Lv, Bing-Rong Gao

**Affiliations:** ^1^State Key Laboratory of Integrated Optoelectronics, College of Electronic Science and Engineering, Jilin University, Changchun, China; ^2^Department of Dental Implantology, School and Hospital of Stomatology, Jilin University, Changchun, China

**Keywords:** bioinspired surfaces, smart surfaces, switchable wettability, fabrication, applications

## Abstract

The surface wettability of plants exhibits many unique advantages, which enhances the environmental adaptability of plants. In view of the rapid development of responsive materials, smart surfaces have been explored extensively to regulate surface wettability through external stimuli. Herein, we summarized recent advancements in bioinspired surfaces with switchable wettability. Typical bioinspired surfaces with switchable wettability and their emerging applications have been reviewed. In the end, we have discussed the remaining challenges and provided perspective on future development.

## Introduction

**Figure 1:**
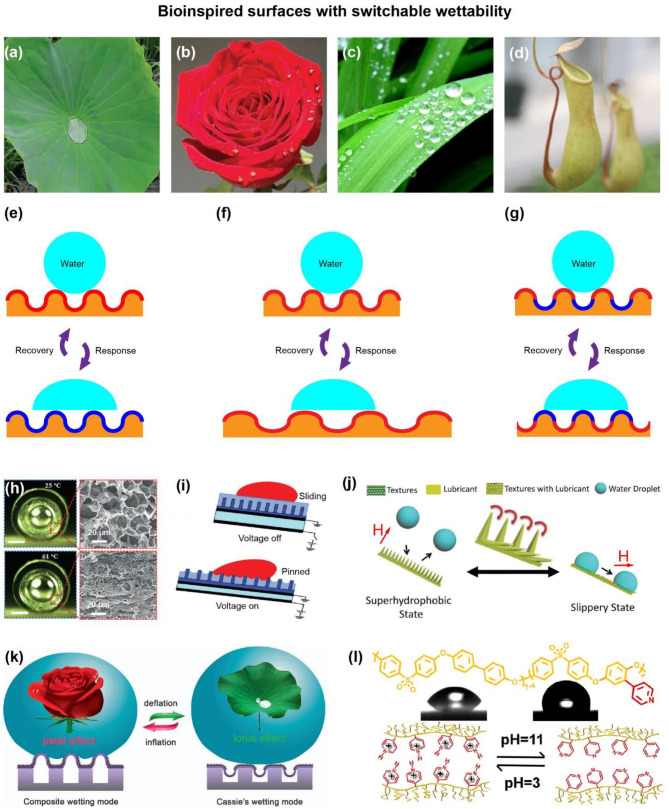
Bioinspired Smart Surfaces with Switchable Wettability. **(a)** Photograph of a lotus leaf. Reproduced from Wang et al. (2018b) with permission of WILEY-VCH. **(b)** Photograph of a rose. Reproduced from Wang et al. (2018b) with permission of WILEY-VCH. **(c)** Photograph of reed leaves. Reproduced from Jiang et al. (2018) with permission of American Chemical Society. **(d)** Photograph of *Nepenthes* pitcher plants. Reproduced from Huang et al. (2017) with permission of WILEY-VCH. The schemes for typical surfaces with switchable wettability based on **(e)** surface chemistry changing, **(f)** surface roughness changing, and **(g)** a combination changing of surface chemistry and surface roughness. **(h)** Photothermal response. Reproduced from Geng et al. (2018) with permission of WILEY-VCH. **(i)** Electric response. Reproduced from Oh et al. (2018) with permission of WILEY-VCH. **(j)** Magnetic response. Reproduced from Huang et al. (2017) with permission of WILEY-VCH. **(k)** Pneumatic response. Reproduced under the terms of the CC-BY Creative Commons Attribution 4.0 International License (http://creativecommons.org/licenses/by/4.0/) (Wang et al., 2018a). Copyright 2018, the authors, published by Springer Nature. **(l)** PH response. Reproduced from Zhu et al. (2017) with permission of American Chemical Society.

The surface wettability of plants exhibits unique advantages (Wu et al., 2009, 2010, 2011a,b; Jiang et al., 2011), which enhances the environmental adaptability and improves survival chances. (Zhang et al., 2012a, 2019b, 2020; Yong et al., 2017) For example, water droplets roll freely on the surface of lotus leaves, which shows self-cleaning characteristics (Figure 1a; Zhang et al., 2012c). Rose petals demonstrate water droplets pinning effect, which is helpful for keeping rose petals hydrated (Figure 1b; Zhang et al., 2012b) Water droplets on the surface of reed leaves prefer to flow along the direction of the parallel leaf veins (Figure 1c; Wang et al., 2015) This anisotropic rolling characteristic plays an important role in collecting dewdrops on the roots and improves the environmental adaptability in dry and hot climates (Jiang et al., 2016). Insects easily slide from the edge of pitcher plants to the inner bottom and provide nourishment for the pitcher plants (Figure 1d; Huang et al., 2017; Zhang et al., 2017). Similar to insects, water droplets are also easy to slide on the liquid-infused surface (Yong et al., 2018). In addition, there are plenty of stimulated-responsive creatures on our planet. For example, organisms show reversible deformable body postures under environment stimulate (Cui et al., 2019). Chameleon owns excellent camouflage capabilities (Jiang et al., 2019b). Venus Flytrap generates closure motions under external forces (Le et al., 2019). Pinecone opens in dry environment and closes in wet environment (Mulakkal et al., 2018). Currently, motivated by such examples with extreme wettability and stimulated-responsive creatures, bioinspired surfaces with switchable wettability have been proposed and prepared (Xin et al., 2018; Jiang et al., 2019a; Han et al., 2020; Li et al., 2020).

Smart surfaces have attracted considerable interests because the surface chemistry and surface roughness play an important role in controlling surface wettability (Fang et al., 2010; Xu et al., 2013; Huang et al., 2017; Wei et al., 2017). Nowadays, the rapid development of responsive materials has enabled surface chemistry and surface roughness change to switch surface wettability through external stimuli (Xin et al., 2016; Wu et al., 2017; You et al., 2018; Salter and Booth, 2019; Zhang et al., 2019c; Fu et al., 2020; Zou et al., 2020). Due to the reversible dynamic control capability, tremendous effects have been devoted to developing driving techniques (Yin et al., 2018; Liu et al., 2020; You et al., 2020) such as temperature, light, electric/magnetic fields, chemicals, and mechanical motion (Han et al., 2015a,b, 2016; Yong et al., 2015). Importantly, the stimulated-responsive bioinspired surface has great prospects in diverse applications, such as droplet manipulations, oil-water separation, cell culture, smart skin (Yang et al., 2018; Liu et al., 2019; Lu et al., 2019).

In this minireview, we focus on the recent advancements in bioinspired stimulated-responsive surfaces with switchable wettability. Typical examples, such as thermal/photothermal, electric, magnetic, mechanical motion and chemical response surfaces, have been summarized. Finally, the challenges and future perspective for smart surfaces with switchable wettability are also discussed.

## Mechanism

Typically, surface chemistry and surface roughness play important roles in the surface wettability. According to the Cassie equation (Li et al., 2019; Zhang et al., 2019a; Namdari et al., 2020): cos θ^*^ = *f*_s_ cos θ_s_ - *f*_a_; *f*_s_ + *f*_a_ =1, θ^*^ and θ_s_ are the apparent contact angle (CA) and intrinsic CA of the substrate. *f*_s_ and *f*_a_ are apparent area fractions of the substrate and air troughs. Therefore, the surface wettability becomes switchable when the surface chemistry and surface roughness change under different environment stimuli. For example, as shown in the Figure 1e, the surface is initially hydrophobic without stimuli because of the structured substrate. Under stimuli, the surface chemical composition changes, leading to the changing of θ_s_. Therefore, the surface water CA changes. Similarly, as shown in the Figure 1f, the surface water CA changes when the apparent area fractions of the substrate and air troughs change (*f*_s_ and *f*_a_) under environment stimuli. In addition, a combination change of surface chemistry and surface roughness can be used to design surfaces with switchable wettability (Figure 1g) because of the combination change of θ_s_, *f*_s_, and *f*_a_.

## Surfaces With Switchable Wettability

### Thermal/Photothermal Response

Benefiting from temperature responsive shape memory polymer (SMP) materials, Cheng et al. have successfully developed thermal response surfaces that tune superhydrophobic characters between isotropic and anisotropic state (Cheng et al., 2018). The groove structure is prepared by heat-pressing a template on the micro/nanostructured pillars surface. The collapsed pillars would recover to the initial structure when the surrounding temperature is above the T_g_. In particular, this thermal response surface shows outstanding rewritable capability. Besides, Geng et al. have made intensive efforts to develop a photothermal responsive tube based on PDMS/rGO-PNIPAm (Figure 1h; Geng et al., 2018). rGO converts light into heat. PNIPAm shows the reversible hydrophilic/hydrophobic switch. Therefore, the PDMS/rGO-PNIPAm tube can be used as an amazing sunlight-driven water transporter by gradient in the surface wettability.

### Electric Response

In 2017, Wei et al. fabricated electric-responsive polypyrrole (Ppy) arrays (Wei et al., 2017). The Ppy array shows reversible morphological transition between hydrophobic nanotubes and hydrophilic nanotips. The morphological transition is because of the volume change of Ppy under different voltage. As a result, the water CAs of the Ppy array are 105 ± 15° under −0.8 V and 44 ± 10° under 0.5 V, respectively. Besides, Oh et al. demonstrated dielectric elastomer-actuated liquid-infused poroelastic film (Figure 1i; Oh et al., 2018) The elastomeric film contracts in the thickness direction and expands in-plane under voltage. Therefore, the liquid-infused poroelastic film can be used for droplet manipulations including droplet oscillation, jetting, mixing.

### Magnetic Response

Magnetically transformable surface was constructed by conformally infusing a liquid lubricant into magnetically responsive hierarchical micropillars (Figure 1j; Huang et al., 2017) The surface shows superhydrophobic property when micropillars are perpendicular to the surface. Whereas, the surface shows slippery property when micropillars are parallel to the surface. This liquid-infused magnetism responsive surface shows adaptive liquid repellency. Besides, due to the switching wetting state, the magnetic response surface can be used for fog harvesting and liquid transport. Similarly, various magnetic response switching wetting surfaces have been successfully designed and fabricated based on PDMS@cobalt microparticles and PDMS@Fe_3_O_4_ (Cao et al., 2017; Li et al., 2018). Recently, Jiang et al. have developed magnetic response Janus microplates arrays (Jiang et al., 2019b). The microplates were prepared by casting a mixture of PDMS and magnetic particles into a polystyrene mold. Then, the one side of microplates was modified by superhydrophobic spray to obtain superhydrophobic property. Another side of microplates was scanned by femtosecond laser to expose the hydrophilic carbonyl irons particles.

### Mechanical Motion

Wang et al. developed a superhydrophobic PDMS skin that switches between lotus leaf and rose petal modes (Wang et al., 2018b). The superhydrophobic PDMS skins with monolithic and hierarchical structures were fabricated by direct laser writing technique. The water droplet rolled off with a slight tilt under ε <50% (rolling state). Whereas, the water droplet firmly stuck on the surface under ε >70% (pinning state). This smart surface shows potential in droplet manipulations by movable joints. Similarly, this group developed pneumatic surfaces by embedding micro-air-sac network in an elastomer (Figure 1k; Wang et al., 2018a). The surface exposes one surface and hiding the other by deflation and inflation.

### Chemical Response

Zhu et al. prepared polyphenylsulfone-pyridine (PPSU-Py*x*)-based nanoporous membrane with switchable wettability in response to pH (Figure 1l; Zhu et al., 2017) Due to the conformational switch of pyridine pendants, the porous membrane shows reversibly switch wettability. The CAs of PPSU-Py*x* are 63.3° in acid solution (*pH* = 3) and 106.5° in alkaline solution (*pH* = 11). Besides, Liu et al. developed chemical response structured copper surfaces by exchanging counterion from PFO^−^ to Cl^−^ (Liu et al., 2018). After the PFO^−^ treatment, structured copper surfaces was filled with fluorine-containing groups, leading to hydrophobic characters. Whereas, the hydrophobic surface loses fluorine-containing groups after the Cl^−^ treatment. As a result, the hydrophobic surface become hydrophilic.

## Conclusion and Outlook

In this minireview, we have summarized the typical stimulated-responsive surfaces with switchable wettability including thermal/photothermal, electric, magnetic, mechanical motion and chemical response surfaces. Taking advantage of the stimulated-responsive characters, the smart surfaces can be used as droplet manipulators, fog collection, smart skin, stem cell differentiation, and others. The further trend of smart surfaces with switchable wettability may be developed from new driving mechanism, fabrication methods, and broaden the application areas. We believe that continued efforts to smart surfaces with switchable wettability would have potential applications in the fields of bionic manufacturing, electronic information, biomedicine, etc.

## Author Contributions

All authors listed have made a substantial, direct and intellectual contribution to the work, and approved it for publication.

## Conflict of Interest

The authors declare that the research was conducted in the absence of any commercial or financial relationships that could be construed as a potential conflict of interest.
